# Scientific issues of zinc‐bromine flow batteries and mitigation strategies

**DOI:** 10.1002/EXP.20220073

**Published:** 2023-07-20

**Authors:** Masud Rana, Norah Alghamdi, Xiyue Peng, Yongxin Huang, Bin Wang, Lianzhou Wang, Ian R. Gentle, Steven Hickey, Bin Luo

**Affiliations:** ^1^ Australian Institute for Bioengineering and Nanotechnology (AIBN) The University of Queensland Brisbane Queensland Australia; ^2^ School of Chemistry and Molecular Biosciences Faculty of Science The University of Queensland Brisbane Queensland Australia; ^3^ Department of Chemistry, Faculty of Science Imam Mohammad Ibn Saud Islamic University (IMSIU) Riyadh Saudi Arabia; ^4^ CAS Key Laboratory of Nanosystem and Hierarchical Fabrication, CAS Center for Excellence in Nanoscience National Center for Nanoscience and Technology Beijing P. R. China; ^5^ School of Chemical Engineering The University of Queensland Brisbane Queensland Australia; ^6^ Redflow Limited Brisbane Queensland Australia

**Keywords:** energy storage, flow battery, functional materials

## Abstract

Zinc‐bromine flow batteries (ZBFBs) are promising candidates for the large‐scale stationary energy storage application due to their inherent scalability and flexibility, low cost, green, and environmentally friendly characteristics. ZBFBs have been commercially available for several years in both grid scale and residential energy storage applications. Nevertheless, their continued development still presents challenges associated with electrodes, separators, electrolyte, as well as their operational chemistry. Therefore, rational design of these components in ZBFBs is of utmost importance to further improve the overall device performance. In this review, the focus is on the scientific understanding of the fundamental electrochemistry and functional components of ZBFBs, with an emphasis on the technical challenges of reaction chemistry, development of functional materials, and their application in ZBFBs. Current limitations of ZBFBs with future research directions in the development of high performance ZBFBs are suggested.

## Graphical abstract

Zinc‐bromine flow batteries are a type of rechargeable battery that uses zinc and bromine in the electrolytes to store and release electrical energy. The relatively high energy density and long lifespan make them an ideal choice for grid‐scale energy storage applications.

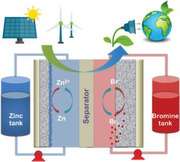

## 1 INTRODUCTION

Energy storage systems have become one of the major research emphases, at least partly because of their significant contribution in electrical grid scale applications to deliver non‐intermittent and reliable power.^[^ ^1^ ^]^ Among the various existing energy storage systems, redox flow batteries (RFBs) are considered to be realistic power sources due to their scalability, high efficiency and long‐life cycles.^[^ ^2^ ^]^ Many types of RFBs based on different redox couples/reactions have been developed, such as iron/chromium (Fe/Cr),^[^ ^3^ ^]^ bromine/polysulfide,^[^ ^4^ ^]^ vanadium,^[^ ^5^ ^]^ and zinc‐bromine.^[^ ^6^ ^]^ The Zinc‐Bromine flow batteries (ZBFBs) have attracted superior attention because of their low cost, recyclability, large scalability, high energy density, thermal management, and higher cell voltage.^[^ ^7^ ^]^

In the early 1970s, the Exxon developed the ZBFB as a hybrid flow battery system, where the energy is stored by plating solid zinc on the anode during charging. As a result, the energy output of the ZBFBs is dependent on the anode surface area and the overall size of the electrolyte storage reservoirs. Unlike other types of flow batteries which rely only on changes of redox states in a single phase, the energy ratings of the ZBFBs are not fully decoupled. After a few decades of development, ZBFBs have been commercially deployed and successfully adapted for the kW to MW scale applications.^[^ ^8^ ^]^ As examples, Ensync Energy deployed a 500 kWh ZBFBs assembly for microgrid scale applications at Illinois Institute of Technology, USA. A ZBFB system of 0.5 MW/3 MWh was deployed by Vionx Energy (was Premium Power, previously) in 2016 in Massachusetts, USA.^[^ ^9^ ^]^ Redflow International Pty Ltd has installed 2053 MWh energy capacity to date through 180 active deployments of ZBFBs across the world.^[^ ^10^ ^]^ Redflow installed a 2 MWh system in California and Primus Power's installed 1 MWh of energy storage in south Africa.^[^ ^11^ ^]^

So far, although great success has been achieved for ZBFBs, nonetheless there are still some practical issues requiring further attention to improve the performance.^[^ ^12^ ^]^ For example, diffusion of aqueous Br_2_ may cause self‐discharge and the relatively slow kinetics of the polybromide conversion reactions are adverse to high power density.^[^ ^13^ ^]^ The solid zinc deposited on the negative electrode can potentially cause a dendrite which penetrate through the separators and lead to short‐circuits. Moreover, it is important to control the pH of electrolyte with suitable acid‐base balance.^[^ ^14^ ^]^ Too low pH causes zinc corrosion that significantly consumes protons in the electrolyte which causes self‐discharge in the ZBFB.^[^ ^15^ ^]^ On the other hand, high pH (≥4) will result in poor zinc deposition and the generation of bromate product like solid Zn (OH)_2_/ZnO,^[^ ^16^ ^]^ which cause membrane clogging. More details of the key performance indicators of ZBFBs as compared to other commercialised flow battery technologies is shown in Table 1.^[^ ^16^, ^17^ ^]^ In this review, we discuss the possible detrimental causes of ZBFBs and their possible solutions which will attract the broad audiences in this area.

**TABLE 1:** Key performance indicators of the current flow battery technologies.

Flow battery technology	All‐vanadium flow battery	Zinc‐bromine flow battery	All‐iron flow battery
Redox chemistry	Positive: VO_2_^+^/VO^2+^ Negative: V^2+^/V^3+^	Positive: Br_2_/Br^−^ Negative: Zn/Zn^2+^	Positive: Fe^2+^/Fe^3+^ Negative: Fe/Fe^2+^
Nominal voltage (V)	1.26	1.85	1.21
Flow type	All‐flow	Hybrid	Hybrid
Energy efficiency (EE%)	∼60–86%	∼70–80%	∼70–75%
Cycling life	>20,000 (VSUN Energy)	Warranted electrode stack lifetime 36,500 kWh energy delivered or 10 years (Redflow)	>20,000 >25 years design life (ESS Inc.)
Major manufacturers	VSUN Energy in Australia Avalon Battery, Vionx, UniEnergy Technologies and Ashlawn Energy in the United States Rongke Power & Prudent Energy in China Sumitomo in Japan redT in Britain	RedFlow Limited in Australia EnSync & Primus Power in the United States Smart Energy & ZBEST Power in China	ESS Inc., United States

## 2 MECHANISM OF ZBFBS

**FIGURE 1:**
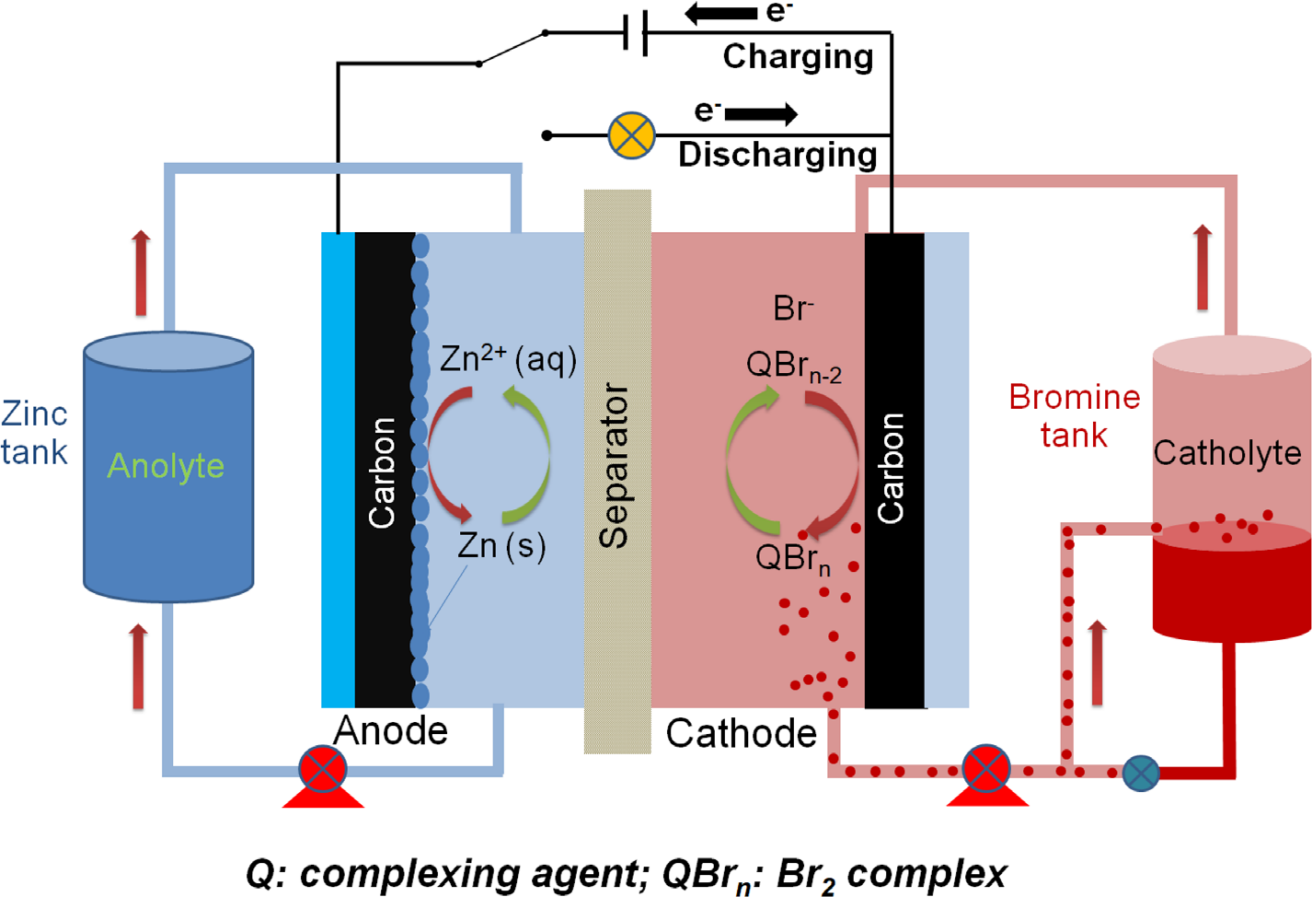
Schematic diagram of the typical ZBFB with different functional components.

The conventional ZBFB contains a negative electrode (Zinc) and positive electrode (bromine) separated by a microporous separator in a single cell. Two tanks of aqueous electrolyte solutions contain the electrochemically active zinc (Zn^2+^) and bromide (Br^−^) species (Figure 1) as well as additional salts like KCl and ZnCl_2_ for good conductivity, and a complexing agent to hold the bromine produced during the charging process in a solution.^[^ ^18^ ^]^ The electrolyte solutions, which have the same initial composition in both tanks, are circulated over the surfaces of both electrodes by the pumps and electrochemical energy is stored/taken out released during the state of charge and discharge. During charging, ionic Zn^2+^ receive electrons from the external circuit and metallic zinc is plated on the negative electrode, whereas bromide ions (Br^−^) release electrons and form Br_2_ at the positive electrode. The opposite reactions occur during the discharge process.^[^ ^19^ ^]^ Figure 1 shows a schematic of typical single‐cell ZBFB consisting of electrodes with corresponding carbon‐based current collectors for the zinc and bromine redox reactions and coupled to two pumps and electrolyte tanks. The microporous separator between the two electrode surfaces maintains ion diffusion during charge/discharge and avoids unwanted short‐circuits in the cell.^[^ ^20^ ^]^ The microporous separator also reduces the Br_2_ passage to the anode to avoid direct chemical interaction with the anode and associated self‐discharge. The basic electrochemical reactions of the zinc bromine battery can be simply represented as follows (reactions 1–5).

Negative electrode:

(1) $$\begin{array}{ccc} & & Zn^{2+}+2e^{-}\to Zn^{0}\left(\mathrm{Charging}\right)\\ & & \left(E_{v}=-0.763/-0.760V\;\mathrm{vs}\;\mathrm{SHE}\;\mathrm{at}\;25{}^{o}C/50{}^{o}C\right) \end{array}$$

(2) $$Zn^{0}\to Zn^{2+}+2e^{-}\left(\mathrm{Discharging}\right)$$

Positive electrode:

(3) $$\begin{array}{ccc} & & 2Br^{-}\to Br_{2}+2e^{-}\left(\mathrm{Charging}\right)\\ & & \left(E_{v}=1.087/1.056V\mathrm{vs}\mathrm{SHE}\mathrm{at}25{}^{o}C/50{}^{o}C\right) \end{array}$$

(4) $$Br_{2}+2e^{-}\to 2Br^{-}\left(\mathrm{Discharging}\right)$$

The overall electrode reaction:

(5) $$\mathrm{ZnB}r_{2}\left(\mathrm{aq}\right)\Leftrightarrow Zn^{0}+Br_{2}\left(\mathrm{aq}\right)E_{v\mathrm{total}}=1.85V$$

**FIGURE 2:**
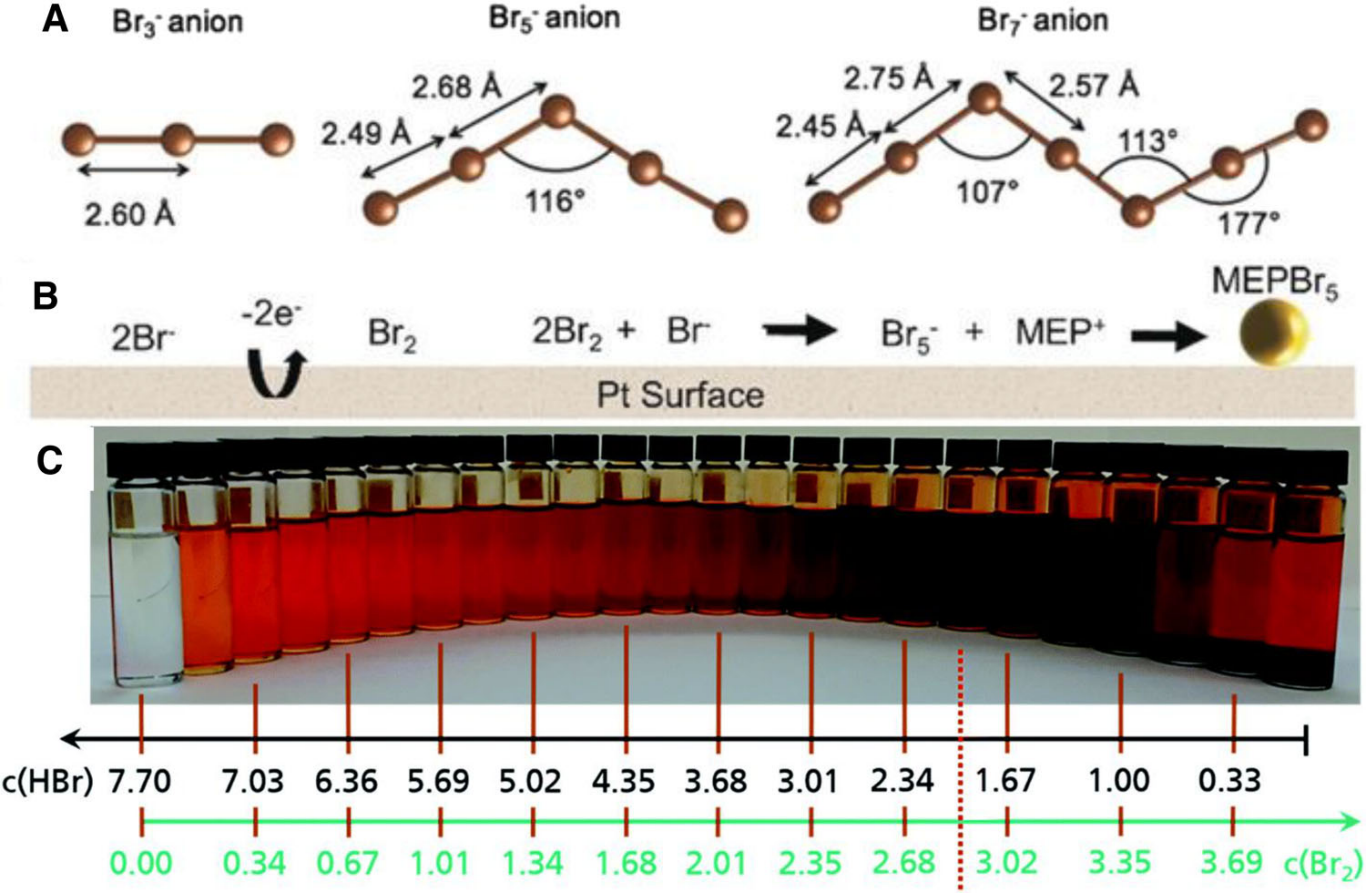
(A) Calculated polybromide structures from Br_3_^−^ to Br_7_^−^ which interact with MEP to form polybromide complexes. (B) Mechanism for the electrochemical formation of MEPBr_5_ on Pt surface. Reproduced with permission.^[^ ^22^ ^]^ Copyright 2019 John Wiley & Sons, Inc. (C) Two phases of the electrolyte as the addition of Br_2_. Reproduced with permission.^[^ ^25^ ^]^ Copyright 2021, the Royal Society of Chemistry.

Apart from the above electrochemical reactions, the behaviour of the chemical compounds presented in the electrolyte are more complex. The ZnBr_2_ is the primary electrolyte species which enables the zinc bromine battery to work as an energy storage system. The concentration of ZnBr_2_ is ranges between 1 to 4 m.^[^ ^21^ ^]^ The Zn^2+^ ions and Br^−^ ions diffuse through the separator to their respective negative and positive half‐cells and flow towards the electrode surfaces during charging. The other species present in the electrolyte flow over the electrodes but do not react at the electrode surfaces. Br_2_ (aq) has a high tendency to diffuse from the positive half‐cell to the zinc metal which causes self‐discharge.^[^ ^13a^ ^]^ Additionally, Br_2_ (aq) has a high vapour pressure (28.26 kPa at 25°C) and can cause environmental and health issues.^[^ ^22^ ^]^ To avoid the diffusion of Br_2_ from the positive to negative half‐cell and to reduce environmental hazards, bromine complexation with different types of quaternary ammonium bromides (QBr) is employed to hold bromine into a complex phase with low vapour pressure.^[^ ^23^ ^]^ The most commonly used QBr complexing agent is *N*‐methylethyl‐pyrrolidinium bromide (MEPBr).^[^ ^22^ ^]^ The Br_2_ is also in equilibrium with the available Br^−^ in the electrolyte and produces polybromide species such as Br_3_^−^_,_ Br_5_^−^, and Br_7_^−^ as shown by the following reactions 6–11 (Figure 2A,B).

(6) $$Br_{2}+Br^{-}\to B{r_{3}}^{-}$$

(7) $$\mathrm{ME}P^{+}+B{r_{3}}^{-}\to \mathrm{MEPB}r_{3}\left(\mathrm{aq}\right)$$

(8) $$2Br_{2}+Br^{-}\to B{r_{5}}^{-}$$

(9) $$\mathrm{ME}P^{+}+B{r_{5}}^{-}\to \mathrm{MEPB}r_{5}\left(\mathrm{oily}\right)$$

(10) $$3Br_{2}+Br^{-}\to B{r_{7}}^{-}$$

(11) $$\mathrm{ME}P^{+}+B{r_{7}}^{-}\to \mathrm{MEPB}r_{7}\left(\mathrm{oily}\right)$$

Bajpal et al. reported that the addition of quaternary ammonium bromide complexes can reduce bromine vapour by almost 100‐fold.^[^ ^24^ ^]^ This quaternary ammonium bromide complex also holds the poly‐bromide species and forms a separate dense layer in solution that reduces the bromine diffusion to the negative electrode.^[^ ^25^ ^]^ The schematic structures of the above bromide species are shown in Figure 2A along with their association with the complex MEPBr. These poly‐bromide complexes are removed from the positive electrode surface by non‐stop circulation of the electrolyte over the surface of the positive electrodes during the charging process. The poly‐bromide complexes settle down in the tank as a separate phase due to their higher density as compared to the aqueous electrolyte. An example of such complex two‐phase electrolyte is shown in Figure 2C. This type of bromine complexation and de‐complexation during discharge require faster electrokinetic activity on the positive electrode.^[^ ^26^ ^]^ A high electrical conductivity and high active surface area are therefore required on the positive electrode to promote the Br_2_ formation and sequestration. Various highly conductive carbon materials with high surface area have been studied and used as bromine electrodes.^[^ ^27^ ^]^

## 3 TECHNICAL CHALLENGES OF PRACTICAL ZBFBS

### 3.1 Hydrogen gas evolution

**FIGURE 3:**
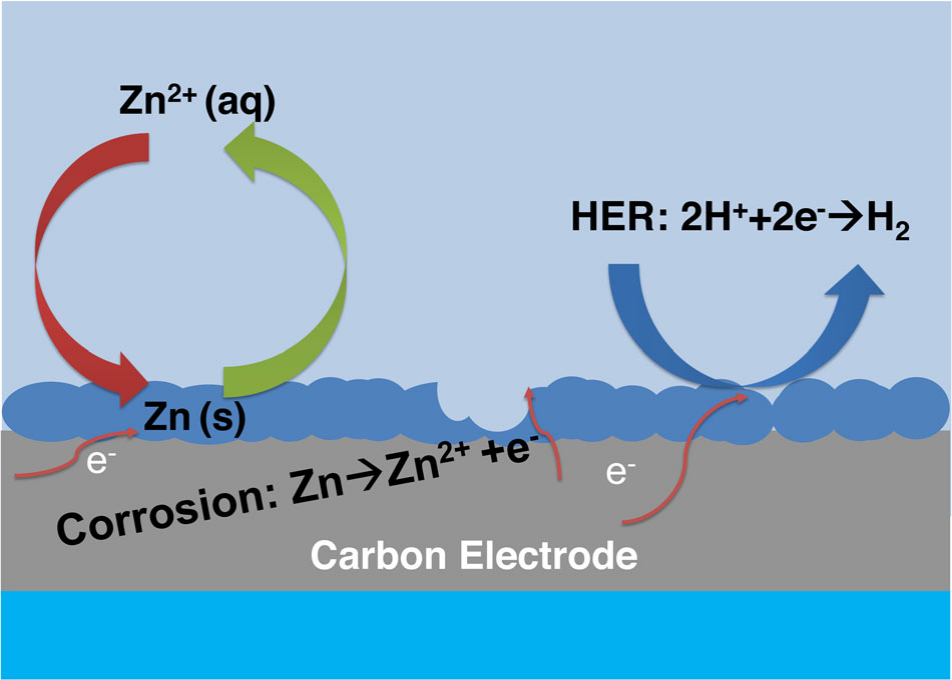
Schematic illustration of the interfacial parasitic reactions on the negative electrode of ZBFBs.

Hydrogen gas evolution is one of the major issues of ZBFBs. This is because hydrogen gas evolution consumes the H^+^ and increases the pH of the electrolyte, which has significant influences on the quality of the deposited zinc on the negative electrode.^[^ ^28^ ^]^ The increased pH eventually leads to the compound of solid zinc oxide or hydroxide and battery failure.^[^ ^29^ ^]^ The ZBFBs are typically maintained to operate between the pH ranges of 1 to 3.5.^[^ ^30^ ^]^ Deposited zinc with moss‐like appearance can be observed in the presence of weakly acidic and basic electrolytes. Hence, it is necessary to maintain the optimal pH range of the electrolyte to have healthy zinc plating in the ZBFB. Typically, a more acidic electrolyte causes significant zinc corrosion which would in turn accelerate hydrogen gas evolution. More hydrogen gas is expected due to electrons being lost (H^+^ + e^−^ **→** H_2_) to protons, rather than being received by the Zn^2+^ on the negative electrode during charging (Figure 3).^[^ ^15^ ^]^ However, the kinetics of such a corrosion reaction are relatively slow compared to the kinetics of zinc deposition, so that zinc can be plated in low pH conditions at high current with coulombic efficiencies of greater than 90 %. In an ideal case, the generated H_2_ (g) would recombine with Br_2_ in the positive half‐cell to generate H^+^ as per the following Equation (12).^[^ ^31^ ^]^

(12) $$H_{2}+Br_{2}\to 2\mathrm{HBr}$$

However, the large quantity of proton reduction in the electrolyte raises the pH and at a certain pH solid Zn(OH)_2_ is formed,^[^ ^8^ ^]^ which may have major consequences on the flow capillary of the ZBFBs. On the other hand, the water (H_2_O) splitting reaction at the high negative electrode potential (−0.83 V) also causes the hydrogen evolution side reaction (HER) during charging. This also results in Zn(OH)_2_ and an increase in pH of the electrolyte.^[^ ^32^ ^]^ Both zinc corrosion and the water splitting process raise the pH of the electrolyte and actively influence the deposition of zinc in the ZBFBs, which is a critical technical issue to be solved. The mechanism of H_2_ gas evolution is more explicitly described in the following section.

#### 3.1.1 Possible reasons for H_2_ gas evolution in ZBFBs

Even though the zinc reduction reaction potential is more negative and therefore thermodynamically more favourable than proton reduction, it is still possible to plate zinc in aqueous acidic solutions. This is due to the high hydrogen overvoltage on zinc that make proton reduction kinetically slow. The H_2_ gas evolution reaction on the negative electrode can be described by a series of chemical and electrochemical reactions. At weakly acidic pH, the H_2_ evolution reaction can occur during charging at a conventional electrode potential (−0.763 V) as follows.^[^ ^33^ ^]^

1. **Volmer reaction (H^+^ (aq) + e^−^ → H (ads))**: This is a typical proton discharge reaction which requires free adsorption sites on the anode to form H (ads). The adsorption sites can be available for this reaction because of moss‐like porous structure of the zinc deposit. This reaction is the so called Volmer reaction where aqueous protons are adsorbed in the pores of moss‐like metal surface and electrochemically reduced to atomic hydrogen.

2. **Tafel or combination reaction (H (ads) + H (ads) → H_2_))**: This chemical reaction is called as Tafel or combination reaction which forms molecular hydrogen by combining two atomic hydrogens. Again, the available porous sites in the metal zinc promote this Tafel reaction.

3. **Heyrovsky reaction (H^+^ (aq) + H (ads) + e^−^ → H_2_))**: This reaction is called as the Heyrovsky (ion +atom) reaction, which combines a proton and atomic hydrogen to form molecular hydrogen.

The strength of the metal—hydrogen bond formed in step (1), determines the rate at which it can be formed or broken during reactions (2) and (3). Since this varies for different metal surfaces, the rate of the HER is a function of the quality of the electrode material. Because of these chemical and electrochemical reactions, H^+^ are consumed in the ZBFB electrolyte and pH tends to rise.

#### 3.1.2 H_2_ generation at different state of charge (SOC)

During discharging/charging, H_2_ gas evolution can occur on the negative electrode which may be responsible for the mossy or spongy like zinc plating (may depends on the state of charge and pH of the electrolyte). The electrolyte pH may have significant impact on the zinc plating and may be responsible to produce mossy zinc plating on the negative electrode. It was claimed that mossy zinc may appears when the pH of the electrolyte approaches to 4.0.^[^ ^30b^ ^]^ This research further claimed that the pH from failed batteries was found to be high ≥3.7 which had mossy zinc plating also unlike other good batteries. The electrode degradation (lower electronic conductivity) and large polarization could be potentially related to such poor zinc plating at high pH ≥3.7. A beaker test at open circuit on a zinc bromine cell revealed that H_2_ gas can be produced on the fresh zinc metal electrodes at a rate of 3.2 × 10^−3^ mL h^−1^ cm^−2^ which is equal to 189 mL h^−1^ when 50‐cell battery stacks with an electrode area of 1175 cm^2^. A current rate of 20 mA cm^−2^ was applied across a pair of Zn electrodes, resulting in a H_2_ gas rate of 5.5 × 10^−2^ mL h^−1^ cm^−2^, which is equivalent to 3200 mL h^−1^ for a full‐size 50‐cell battery stack.^[^ ^30b^ ^]^

**FIGURE 4:**
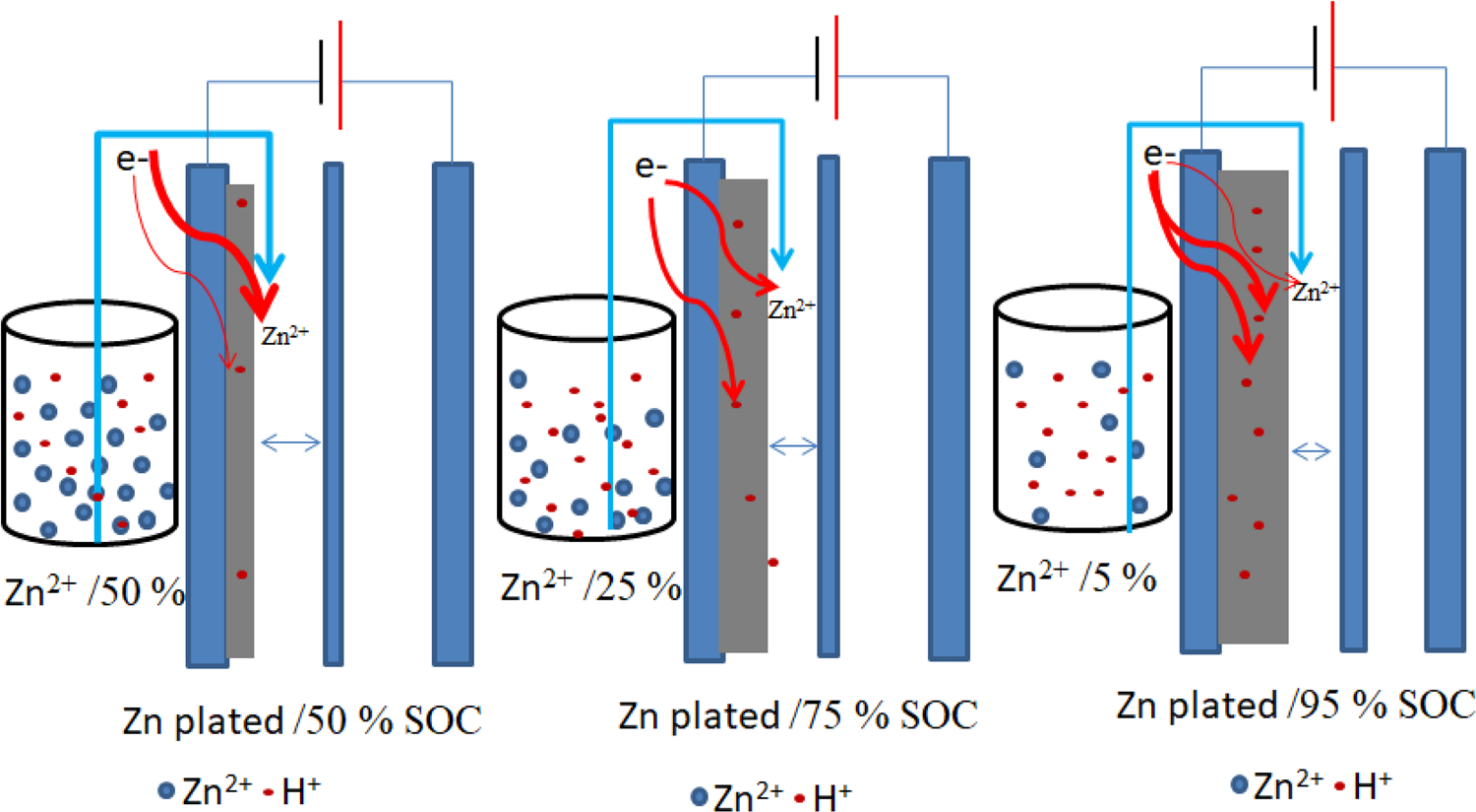
Schematic illustration of three different SOC% of negative electrode, where H^+^ is more likely to be reduced with decreased zinc concentration in the electrolyte.

Basically, during charge, Zn^2+^ ions in solution are reduced and converted to Zn metal. Near the top of charge, depletion of Zn^2+^ leads to concentration polarisation and increase the proportion of the charging current consumed by the H^+^ reduction reaction that leads to H_2_ gas evolution. The hydrogen evolution rate is affected by the quality of the zinc plating. Figure 4 shows an example of different states of charge (50 % SOC, 75% SOC, and 95 % SOC, respectively) of the electrode, where red dots represent H^+^ and blue dots represent Zn^2+^ in the electrolyte. It can be shown that the Zn^2+^ in the electrolyte near the top of charge decreases whereas the H^+^ concentration near the surface of the electrode is always high. As such, H_2_ evolution occurs mostly near the top of charge with mossy or spongy like zinc being plated. Likewise, during discharge, the oxidation reaction occurs, and Zn metal reverts to Zn^2+^. The high surface area of the mossy or spongy zinc deposit, increases the rate of corrosion of the zinc metal and the electrons released are used to reduce protons rather than going through the external circuit to reduce bromine (Figure 4).

#### 3.1.3 H_2_ gas evolution at electrolyte flow imbalance condition

The flow rate is a critical parameter for the ZBFB. The number of ions transported to the anode and cathode surfaces depends on the flow rate of the electrolyte. A certain volume of electrolyte is continuously pumped to ensure the availability of sufficient active ions at the electrode surface to support the charge/discharge reactions in the cell. Stoichiometrically, the total number of moles of zinc plated on the anode should be equal to the number of moles of Br_2_ produced at the cathode during charging. If the flow of the electrolyte is somehow compromised/imbalanced then equal amounts of Zn^2+^/Br^−^ will not be available on the electrode surface and side reactions. At the negative electrode, inadequate supply of Zn^2+^ will lead to concentration polarisation and hydrogen evolution. The H^+^ loss by this mechanism will also allow the pH to be increased and ultimately Zn(OH)_2_/ZnO will be produced leading to flow capillary blockage.

#### 3.1.4 Consequences of H_2_ generation

The H_2_ evolution in Zinc based technology is a very common technical issue due to zinc being thermodynamically unstable in the presence of hydrogen ions. In ZBFBs, the H_2_ evolution can cause uneven and porous zinc plating which may cause unstable coulombic efficiency, dendrite growth and shorten the life span of the batteries. During charging, the negative electrode is operated at reduction potentials of less than −0.76 V to plate Zn on the surface. With polarization, the potential can drop to −0.83 V at which further splitting of H_2_O on the electrode surface increases the rate of H_2_ gas evolution and OH^−^ formation according to the HER illustrated in Figure 3.^[^ ^34^ ^]^

(13) $$\begin{array}{c} 2H_{2}O+2e^{-}\to H_{2}\left(g\right)+2OH^{-}\left(\mathrm{aq}\right)\mathrm{Ev}=-0.83V\;\mathrm{vs}\;\mathrm{SHE} \end{array}$$

This leads to an increase of pH in the electrolyte that could cause Zn^2+^ to precipitate as an oxide or hydroxide. The Zn associated with this solid Zn(OH)_2_ or ZnO is considered to be lost as it is not involved in zinc deposition, having a significant impact on the energy and coulombic efficiencies of the ZBFB. A Sandia report revealed that the ZnO layer formed on the zinc metal is likely produced when zinc metal reacts with water and forms hydrogen gas as a coproduct.^[^ ^30b^ ^]^

(14) $$\mathrm{Zn}+2H_{2}O\Leftrightarrow Zn^{2+}+H_{2}+2OH^{-}$$

(15) $$\mathrm{Zn}+2H_{2}O\Leftrightarrow \mathrm{Zn}{\left(\mathrm{OH}\right)}_{2}+H_{2}$$

(16) $$\mathrm{Zn}+H_{2}O\Leftrightarrow \mathrm{ZnO}+H_{2}$$

These co‐products also precipitates in the electrolyte and increases the chance of clogging of the flow paths; this reduces the flow of the electrolyte.^[^ ^35^ ^]^

### 3.2 Self‐discharge

Thermodynamically, Br_2_ is corrosive to zinc and will cause severe self‐discharge in ZBFBs. The self‐discharge mechanism in the ZBFB is occurred by the reaction between aqueous bromine and zinc metal where bromine is transported through the membrane to the negative half‐cell.^[^ ^36^ ^]^ Therefore, it is crucial in the ZBFB system to minimize the diffusion of Br_2_ to the Zn. The Br_2_ transport rate can be measured from the H‐cell test simply by placing a well‐mixed standard electrolyte solution in one side of a separator and same solution without Br_2_ on the other side.^[^ ^37^ ^]^ The transport rate can be calculated by measuring the bromine concentration on the other side which can be denoted in terms of a pseudo‐current. The measured Br_2_ transport rate (mol of Br_2_/cm^2^ × second) is responsible for equivalent current loss and can be calculated as follows.^[^ ^30b^ ^]^

Mol of Br_2_/cm^2^‐second = (area of the membrane × second) × (2 equiv./mol of Br_2_) × (26.8 Ah/equiv.) (17)

### 3.3 Dendrite growth/zinc morphology

**FIGURE 5:**
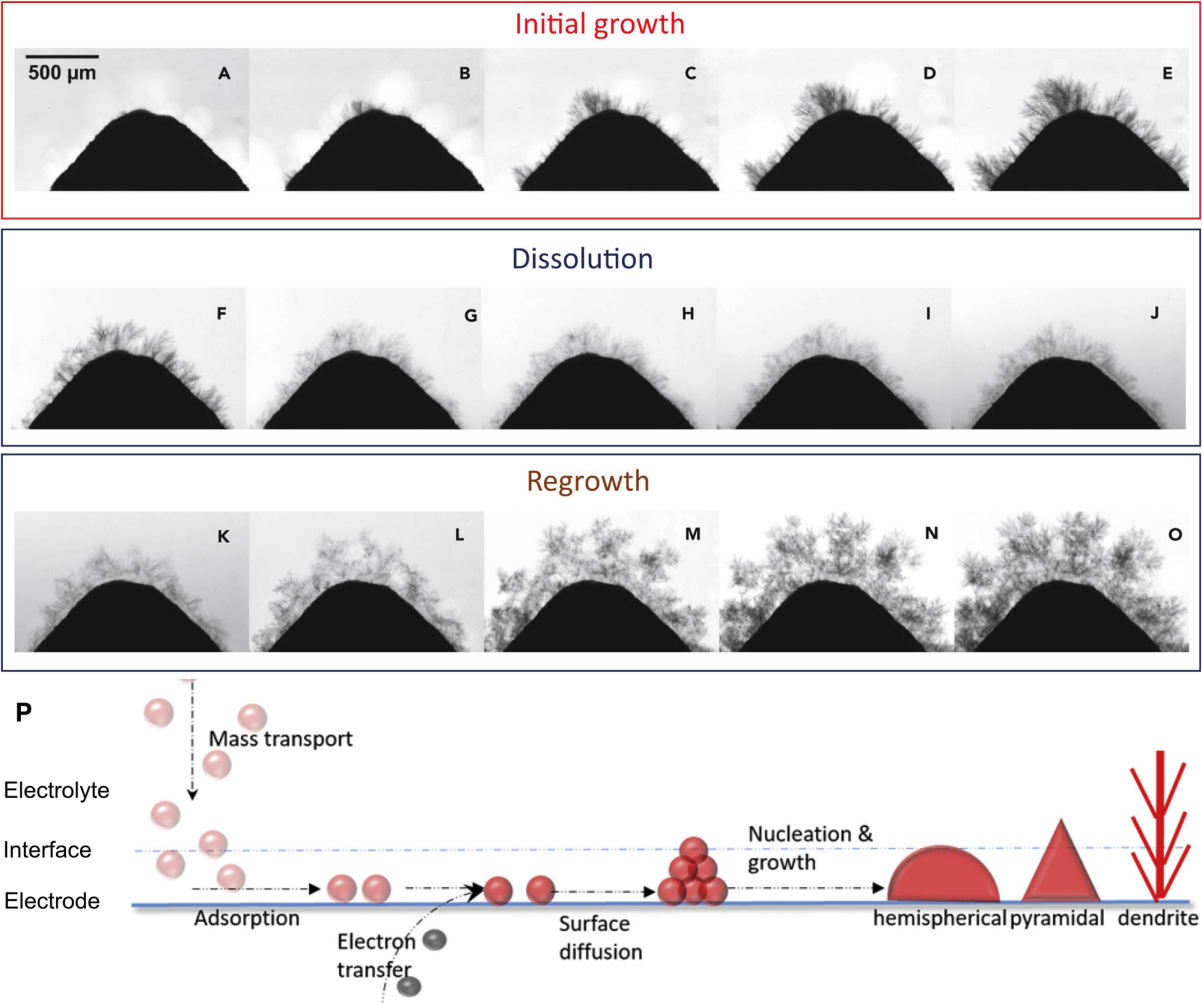
Dendrite formation on zinc (A–E), dendrite dissolution (F–J), and dendrite regrowth (K–O) at different stages of charge and discharge. Reproduced with permission.^[^ ^79^ ^]^ Copyright 2018 Elsevier Inc. (P) Artistic mechanism to grow dendrite in ZBFBs. Reproduced with permission.^[^ ^39^ ^]^ Copyright 2020, the royal Society of Chemistry.

Like other metal‐based rechargeable batteries, dendrite growth on the zinc electrode during charging/discharging is another critical issue in ZBFBs. Dendrite formation is the consequence of uneven current density and non‐uniform electric field on the localized area of the zinc surface.^[^ ^38^ ^]^ It is a cumulative process throughout the cycles with steps which can be categorised into initial growth, dissolution, and regrowth as shown in Figure 5.^[^ ^39^ ^]^ Basically, the residual dendrites from the previous cycles act as deposition sites for subsequent cycles, leading to denser dendrites. Due to the non‐uniform deposition of the zinc, the electric field in this region becomes higher and consumes more Zn^2+^ to form dendrite. As the dendrites continue to grow, they can penetrate the membrane and cause short circuiting. Dendrite formation also decreases battery efficiency and potentially causes flow channel blockage. Short circuits can also lead to internal heating with a potential for battery fires. The flow rate of the electrolyte to the electrode, zinc plating thickness and current density all influence the dendrite growth. Therefore, deep understanding on the fundamental of the plating/stripping process at the zinc anode is crucial for ZBFBs to mitigate the uneven deposition of zinc on the electrode.^[^ ^40^ ^]^

### 3.4 Shunt current loss

**FIGURE 6:**
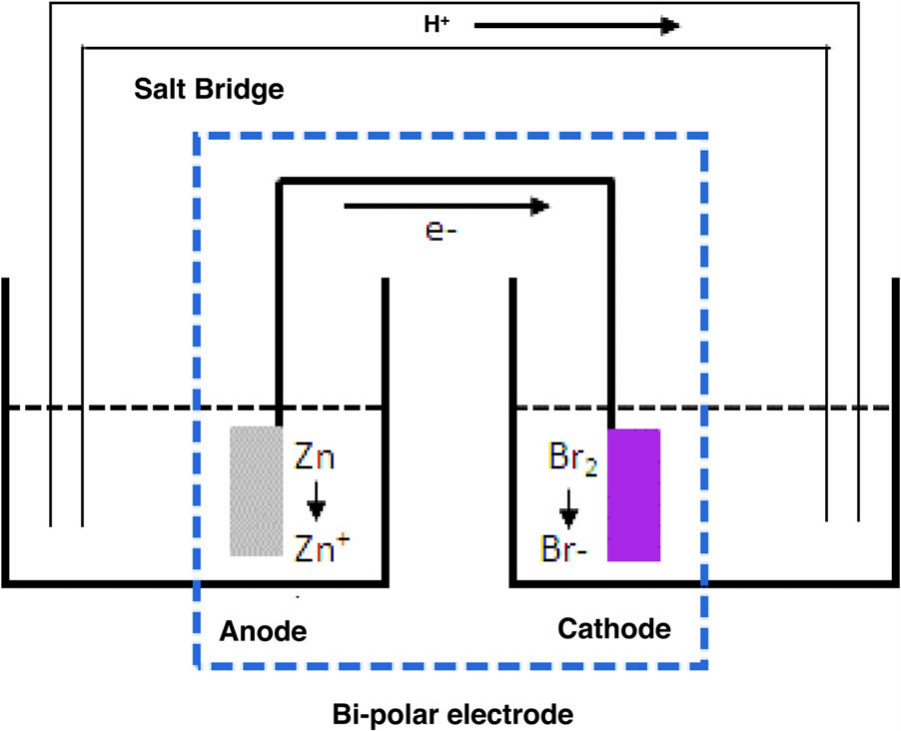
Two half cells at closed circuit to demonstrate the shunt current mechanism. Reproduced with permission.^[^ ^42^ ^]^ Copyright 2016, John Wiley & Sons, Inc.

Multiple single cells are configured in series by bi‐polar electrodes which are employed between adjacent cells in a large ZBFB stack. The inlet and outlet flow manifolds are configured to flow and collect electrolyte continuously where the battery stack shares a common electrolyte path. As the electrolytes are ionically conductive, the manifolds and bi‐polar cells carry some current which cause current loss so called shunt current loss. This shunt causes unwanted side reactions including corrosion and gas generation which has significant influence on the electrolyte pH and zinc plating.^[^ ^41^ ^]^ Hence, it is crucial to reduce the shunt current losses by proper manifold and stack design. Due to the bi‐polar electrode the conductive channel between two electrodes is always in a closed circuit at no load and also at loaded conditions of the battery. Therefore, a potential difference always exists across the electrodes which allow the electrons to flow through the electrically conductive path of the bi‐polar electrode while protons can travel through the manifold and flow channels externally from the negative cell to the positive cell to complete the circuit (Figure 6).^[^ ^42^ ^]^ The protons migrate through the external path thus completing the circuit which creates an internal shunt current throughout the stack.^[^ ^42^, ^43^ ^]^

The study of shunt currents in flow batteries was first introduced by NASA in the 1970s. The shunt current loss is dependent on the resistance of the electrolyte, and the diameter of the manifold. The minimum manifold currents, based on a predicted manifold diameter of 0.04 and 0.2 cm, were calculated to be 2 and 48 mA, respectively.^[^ ^13a^ ^]^ Therefore, to reduce the shunt current, it is desirable to have longer channels with smaller cross‐sectional areas which requires more electrolyte pumping power to circulate the electrolyte through the stack. As pumping systems are often driven by the battery itself, a trade‐off between pumping power and shunt loss therefore needs to be optimized in real ZBFB systems to achieve maximum efficiency. However, the use of small holes can lead to difficulties because small holes are likely to plug easily with either carbon particles or second phase particles.

## 4 ADVANCED MATERIALS FOR ZBFBS

To address the existing issues and challenges of ZBFBs, proper materials selection and strategies are key to achieve high performance batteries. Some advanced materials with outstanding properties such as high electrocatalytic activity to promote the redox reaction, high electrical conductivity, and large surface area are promising to solve the above challenges. Over the years, different types of porous materials, polymers, and their composites have been used for electrode design in ZBFBs.^[^ ^44^ ^]^ Also, various ionically conductive additives and bromine complexes in the electrolyte have been explored to achieve faster reaction kinetics. In this review, the technical benefits of the existing materials and their further development to achieve high performance ZBFBs will be discussed.

### 4.1 Electrode materials

#### 4.1.1 Carbon based materials

**FIGURE 7:**
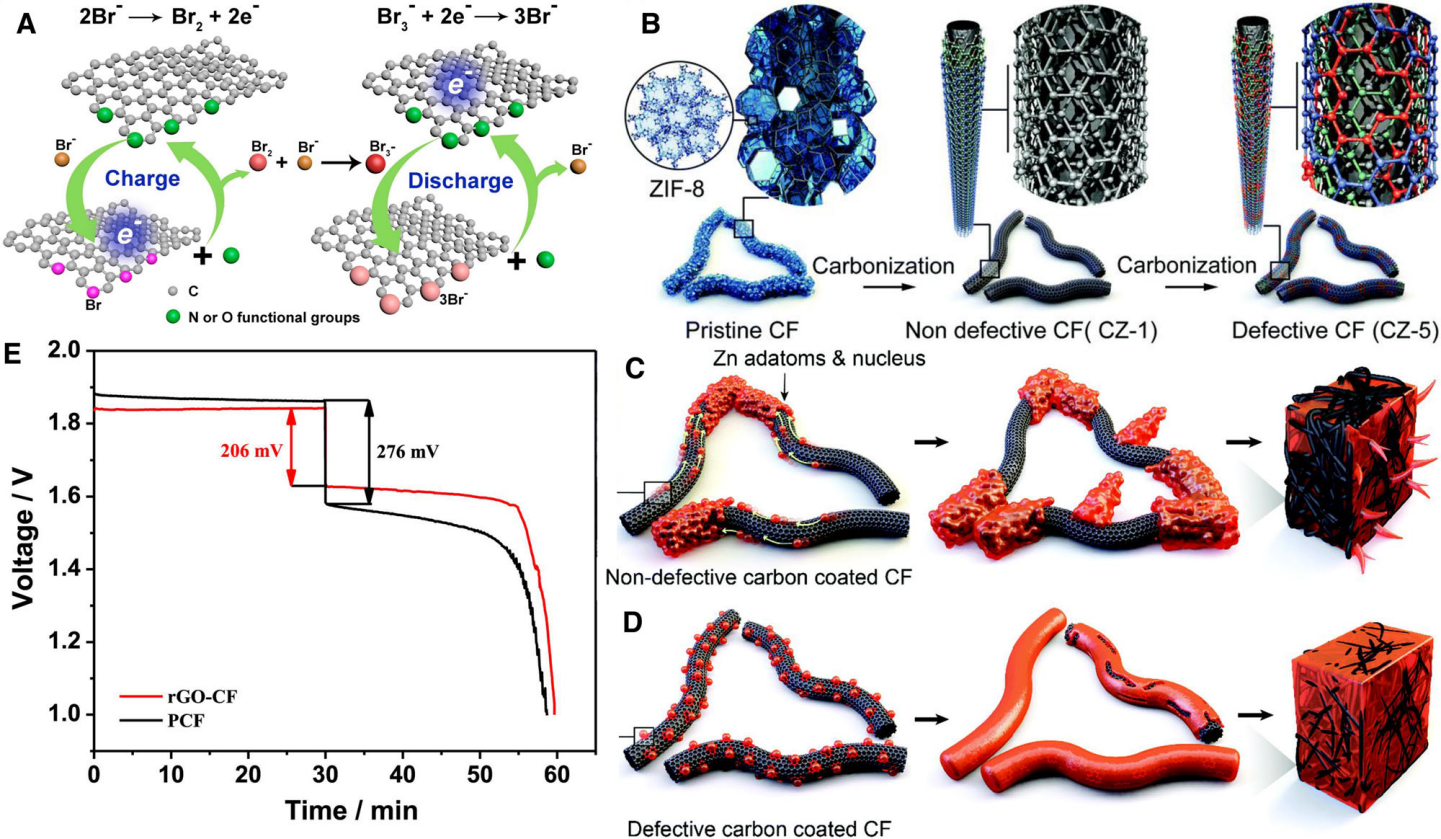
(A) Catalytic mechanism of PNSC during charge/discharge. Reproduced with permission.^[^ ^45^ ^]^ Copyright 2018, Elsevier. (B) ZIF‐8 derived non‐defective and defective carbon layers on the CF surfaces. (C) Zn agglomeration and non‐uniform plating on the non‐defective CF surface and (D) uniform nucleation and lateral plating of Zn on the defective carbon‐coated CF. Reproduced with permission.^[^ ^46^ ^]^ Copyright 2020, The Royal Society of Chemistry. (E) The voltage profile of rGO‐CF in comparison to PCF. Reproduced with permission.^[^ ^47^ ^]^ Copyright 2018, Elsevier.

Carbon based materials have been extensively implemented in all energy storage devices due to their natural abundance, ease of synthesis, low cost, effective surface area for ion exchange and electrically conductive nature (Table 2). Importantly, carbon materials have been tuned with mesoporous, microporous, and macroporous structures for different functions in energy storage systems. Hence, according to the requirements the carbon materials can be used to promote the catalytic activity and mitigate Zinc dendrite in ZBFBs. The catalytic conversion of Br_2_ to Br^−^ and vice versa is crucial to enable high efficiency ZBFBs. Taking this into account Wang et al. demonstrated porous nano‐sheet carbon (PNSC) with fast kinetics for Br_2_/Br^−^ conversion.^[^ ^45^ ^]^ The PNSC was synthesised through the pyrolysis of nano‐sheet zeolite‐type metal organic framework at 900°C under nitrogen with a heating rate 5°C min^−1^ followed by 1200°C in the presence of carbon dioxide. After the pyrolysis, the PNSC provided well‐defined electron conductive frameworks which shortened the electron transport and increased the electronic conductivity and sped up the catalytic mechanism of the Br_2_/Br^−^ redox reaction. Figure 7A represents the catalytic activity of the PNSC during charge/discharge. In addition, the highly porous (∼2085 m^2^/g) PNSC substantially increased the ion diffusion rate within the electrode framework which led the voltage efficiency of 83 % and energy efficiency of 82 % at 80 mA cm^−2^.

**TABLE 2:** Comparison of carbon‐based electrode materials for Zinc–bromine flow batteries.

Electrode Materials	Surface area (m^2^ g^−1^)	CE%	VE%	Current density mA cm^−2^	Max cycles	BSA	Voltage loss (mV)	Separator	Ref.
Carbon based materials									
PNSC	2085	82	83	80	200	n/a	280	Daramic	^[^ ^45^ ^]^
ZIF‐8@CF	n/a	97	69	120	5000	n/a	760	Asahi	^[^ ^46^ ^]^
NiPPC	158	88.3	91.9	100	100	n/a	130	n/a	^[^ ^48^ ^]^
rGO‐3D carbon	n/a	95	85	80		MEP	276	n/a	^[^ ^47^ ^]^
BOMCs	1647	96	82.9	82	200	MEP	370	n/a	^[^ ^49^ ^]^
TPPC	270	99	80	80	100	n/a	230	n/a	^[^ ^50^ ^]^
SWCNTs	n/a	98	71	20	200	MEP: MEM	310		^[^ ^51^ ^]^
SWCNTs (90%)	n/a	72	90	40	n/a	MEP: MEM	190	Daramic	^[^ ^54^ ^]^
PAN		96	83	40	50	MEP	200	Daramic	^[^ ^52^ ^]^
Doped carbon based materials									
NGF	18.5	85	66	5	1000	m/a	n/a	n/a	^[^ ^57^ ^]^
NDGN	n/a	98	80	120	100	n/a	400	n/a	^[^ ^56^ ^]^
NMC	720	100	84	80	200		250	n/a	^[^ ^58^ ^]^
NTCF	n/a	97.2	64.8	180	140	n/a	723	n/a	^[^ ^7a^ ^]^
NDC	n/a	99	83	80	200		250	n/a	^[^ ^53^ ^]^
BDG	n/a	86	73	30	100	n/a	145	Daramic	^[^ ^59^ ^]^
N,O,G	n/a	95	77	40	100	MEP	110	Daramic	^[^ ^60^ ^]^
Composite and Hybrid based materials									
Pt@Graphite	n/a	93	87	30	300	MEP: MEM	100	Daramic	^[^ ^62^ ^]^
MO	n/a	95	83	30	1000	MEP	400	Asahi	^[^ ^63^ ^]^
Sn3DC	n/a	99.4	n/a	100	290	n/a	370	n/a	^[^ ^64^ ^]^
CG	1832	92	89	20	70	MEP	n/a	n/a	^[^ ^65^ ^]^

**FIGURE 8:**
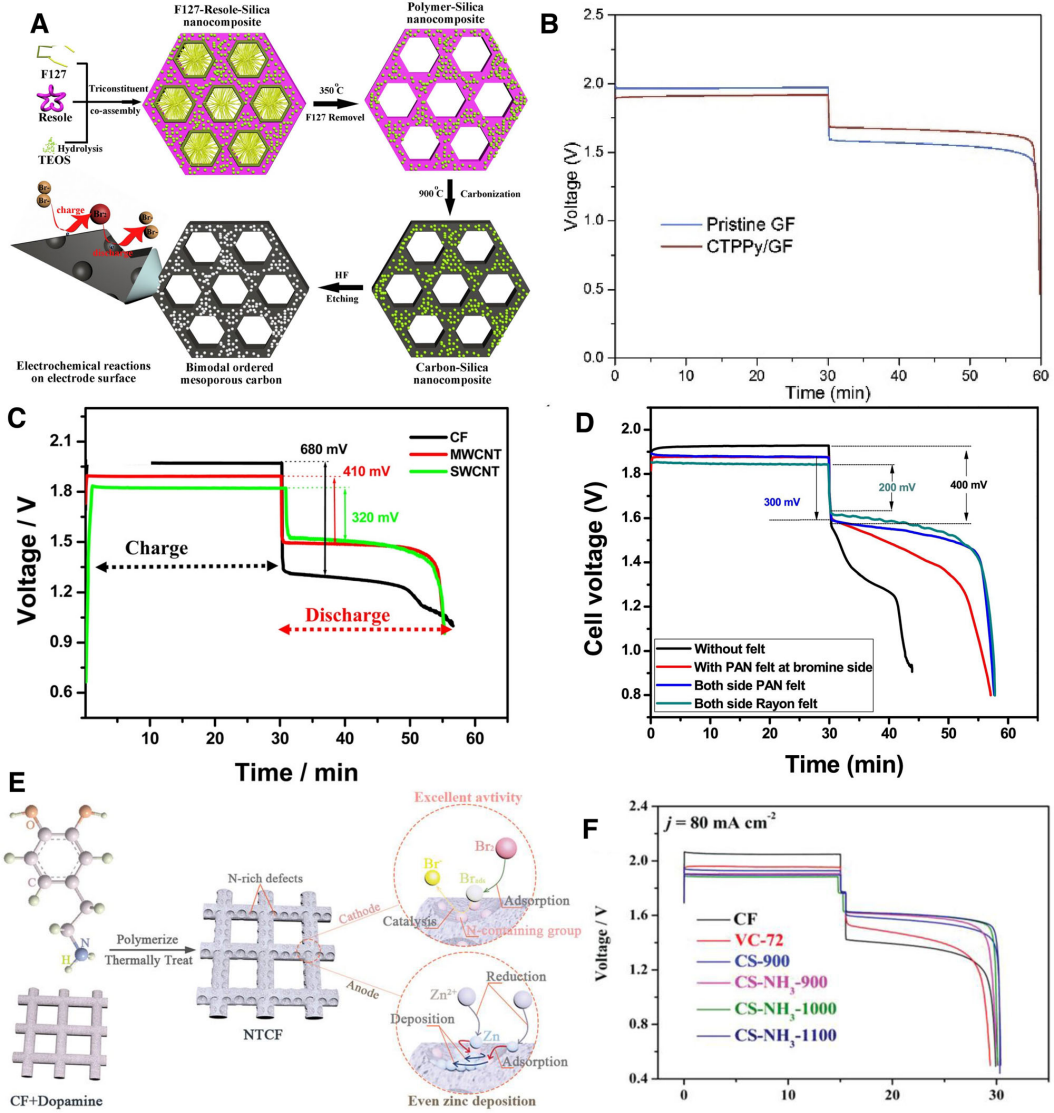
(A) bimodal highly ordered nanostructured carbon to promote Br_2_/Br‐ conversion. Reproduced with permission.^[^ ^49^ ^]^ Copyright 2016, Elsevier. (B) Galvanostatic discharge/charge profiles of ZBFBs with the CTPPy‐modified graphite felt and unmodified graphite felt. Reproduced with permission.^[^ ^50^ ^]^ Copyright 2018, Elsevier. (C) Charge and discharge profiles of CF, MWCNT modified CF, and SWCNT modified CF as positive electrode at the current density of 20 mA cm^−2^. Reproduced with permission.^[^ ^51^ ^]^ Copyright 2014 American Chemical Society. (D) Comparison of the galvanostatic discharge/charge profile of the cell with different cell/electrode configuration at the current of 20 mA cm^−2^. Reproduced with permission.^[^ ^52^ ^]^ Copyright 2018, Elsevier. (E) NTCF electrode with abundant N‐rich defects for ZBFBs. Reproduced with permission from Ref.[7a] Copyright 2021 John Wiley & Sons, Inc. (F) Galvanostatic discharge/charge profiles of different carbon based electrodes at 80 mA cm^−2^. Reproduced with permission.^[^ ^53^ ^]^ Copyright 2019 John Wiley & Sons, Inc.

Zinc dendrite formation in ZBFBs is a demanding topic because of its propensity to cause short‐circuiting and capacity drop due to easy access of dendrites to the Br_2_ while operating the cells. A defective carbon surface was proposed and implemented by Lee et al. as an effective way to mitigate dendrite growth in ZBFBs.^[^ ^46^ ^]^ The zeolitic imidazole framework‐8 (ZIF‐8) was selected as a sacrificial template owing to its flexibility to control the defect structures of the surface. The ZIF‐8 was converted into the porous defective carbon through high temperature treatment (Figure 7B–D). It was proposed that the dangling bonds of the defects are able to prevent the consequent aggregation of Zn. Through this interatomic interaction a defective carbon with smooth Zn plating enabled excellent stability with 97% coulombic efficiency up to 5000 cycles at 100 mA cm^−2^. Figure 8B–D show the defective carbon synthesis process along with the mechanism to produce dendrite free Zn plating on the defective carbon coated CF in comparison to the non‐defective one.

In another approach, a mesoporous carbon material was produced through carbonising the pomelo peel with the presence of nickel salts, which can be washed away by acid afterwards.^[^ ^48^ ^]^ The nickel species acted as a potential catalyst for graphitization and generated pores on the carbon surfaces, both of which improved the electrochemical performance of the battery. The synthesized mesoporous carbon showed a remarkable activity toward Br_2_/Br redox reaction with energy efficiency of 81.2% at 100 mA cm^−2^. More strikingly, the cell introduced 78.5 % energy efficiency at 120 mA cm^−2^.

Reduced graphene oxide (rGO) was introduced into the positive electrode by Suresh et al. to improve the poor reversibility of Br_2_/Br^−^, energy efficiency, rate capability, and cycle longevity.^[^ ^47^ ^]^ The rGO in the positive electrode played an important role in improving the bromine kinetics in ZBFBs. Voltage loss of 276 mV was seen for the pristine materials, whereas the rGO modified electrodes exhibited 206 mV loss indicating the electrochemical kinetics are improved (Figure 7E). The cell exhibited an energy efficiency of 80.75 % at 80 mA cm^−2^.

Bimodal highly ordered mesostructured carbons (BOMCs) were fabricated by an evaporation induced tri‐constituent co‐assembly method to achieve high activity of Br_2_/Br^−^.^[^ ^49^ ^]^ The BOMCs were designed to produce pores with walls of around 2 and 5 nm to adsorb Br_2_ and provide more active sites for the Br_2_/Br^−^ reaction (Figure 8A). The maximum energy efficiency obtained was 80.1 % at 80 mA cm^−2^.

Tubular polypyrrole (CTPPy) with N and O‐containing functional groups was carbonised for 2 h at 800°C under a N_2_ atmosphere at a heating rate of 5°C min^−1^ to achieve nitrogen and oxygen functionalised positive electrode materials for ZBFBs (Figure 8B).^[^ ^50^ ^]^ As a result of the higher electronegativity of N and O dopants, the electronic properties of carbon atoms were modified with improved capability in adsorbing bromide species onto the carbon scaffold (Figure 8B). The BET surface area of this carbon material was 66 m^2^ g^−1^. ZBFBs with the functionalised positive electrode showed less voltage loss and improved energy efficiency of 76% at 80 mA cm^−2^. On the other hand, the battery with unmodified graphene felt exhibited an energy efficiency of only 69.4%.

Electro‐catalytic performance of the positive electrode was further evaluated by the use of single wall and multi‐wall carbon nanotubes.^[^ ^51^ ^]^ These materials have been considered for use as the positive electrode because of their high electrical conductivity, electro‐catalytic activity, and excellent mechanical strength. SWCNTs show lower voltage loss indicating a faster electrokinetic reaction as compared to MWCNTs and CF (Figure 8C). The SWCNTs outperformed the MWCNTs showing energy efficiency of 98 % retention even after 200 cycles.

Carbon nanotubes (CNT) also have been used to modify the glassy carbon to be used as a positive electrode in ZBFBs.^[^ ^54^ ^]^ The voltammetric peak exhibited current density 2.5 times higher than the pristine glassy carbon which demonstrates accelerated electro‐catalytic effect of CNT towards Br^−^/Br_2_ redox reaction. The voltage loss also improved using 90% CNT modified electrode which is a good indication of faster electrochemical kinetics. The 90 % CNT modified electrode showed coulombic efficiency, voltaic efficiency, and energy efficiency as 87%, 77%, and 67%, respectively.

Suresh et al. investigated three different electrodes to achieve high performance ZBFBs.^[^ ^52^ ^]^ Polyacrylonitrile (PAN) and rayon‐based carbon felts (both 3 mm thickness) electrodes were studied and compared with the graphite composite plate (6 mm thickness) to evaluate the electrochemical performance of ZBFBs. Among these three electrodes, the rayon‐based carbon felt exhibited superior electrochemical performance with high coulombic efficiency (96.26%), voltaic efficiency (83%), and energy efficiency (79.4%) at 20 mA cm^−2^. The voltage loss of the ZBFB cell without felt is high (400 mV), whereas it decreased to 200 mV when the carbon felt is introduced on both sides in the cell design (Figure 8D). The rayon felt modified electrode showed a further lower potential drop of 200 mV. This could be ascribed to the many active sites and efficient contact between electrode and electrolyte.

#### 4.1.2 Doped materials

Carbon materials used in electrodes can typically provide high electronic conductivity and surface area. However, the raw carbon materials are typically non‐polar, which do not provide any chemical affinity. The electronic properties of carbon materials can be modulated by various chemical doping with hetero atoms. For example, nitrogen doping in carbon materials modulate the electron for electrocatalytic process because of its higher electronegativity. Specially, the doping of carbons by various heteroatoms have shown significant progress in the field of energy storage such as sulphur, phosphorus, and boron. By the doping process of the carbon materials by such heteroatoms, the chemical affinity of the pristine carbon materials can be improved. Different doped carbon‐based materials are a good choice to improve the electrical conductivity of the electrode and accelerate the electro‐catalytic activity of Br_2_/Br^−^ couples.

For example, when graphene is doped with N, typically three common bonding sites are formed in the carbon structure commonly known as graphitic, pyridinic, and pyrrolic N. N‐doping in graphene can enhance electronic conductivity due to its higher electronegativity (3.07).^[^ ^55^ ^]^ The pyridinic N is considered as electron‐rich donor with an extra pair of electrons into its p‐orbitals. Therefore, naturally it can act as a Lewis base site to interact.^[^ ^56^ ^]^ It has been shown by the DFT calculations that the Br_2_ can only weakly interact with N‐doped graphene. However, the highly concentrated bromide/polybromide species could be strongly adsorbed by the nitrogen doped graphite felt (NGF) which can inhibit Br_2_ crossover diffusion.^[^ ^57^ ^]^ Wu et al. introduced N‐doped graphene nanoplatelets (NDGN) as an effective and promising catalyst for Br_2_/Br^−^ redox reactions in ZBFBs.^[^ ^56^ ^]^ More strikingly, the N‐doped graphene shows almost no capacity degradation after 100 cycles with an energy efficiency of 84.2% at 80 mA cm^−2^.

N‐doped mesoporous carbon (NMC) was investigated as a potential electrode material to improve the electrochemical activity of ZBFBs.^[^ ^58^ ^]^ Firstly, the mesoporous template shows high specific surface area. Secondly, the heteroatom‐activated carbon acted as the active sites exhibited promising electrocatalytic activity towards the Br_2_/Br^−^ redox couple. The NMC with high surface area showed an energy efficiency of 84.3% at 80 mA cm^−2^ with almost no degradation after 200 cycles which could be an indication of no self‐discharge as well.

Recently, Lu et al. introduced N‐rich defects in multifunctional carbon felt (NTCF) which induced high catalytic activity on Br_2_/Br^−^ reactions (Figure 8E).^[^ ^7a^ ^]^ Further, the lower energy barrier of N doping exhibited uniform Zn plating by adsorbing Zn atoms. By using this N‐doped carbon felt in both electrodes, stable ZBFBs were achieved with an unprecedented coulombic efficiency of 97.25% at high current density of 180 mA cm^−2^. The N‐doped carbon felt also ensured less voltage drop, indicating a faster catalytic activity. Xiang et al. experimented an efficient N‐doped carbon felt (NDC) for ZBFBs.^[^ ^53^ ^]^ The resultant carbon exhibited some promising features such as large surface area, high electronic conductivity, and abundant functional groups which benefited the formation and exposure of the active sites toward the Br_2_/Br^−^ redox couple. The N doped carbon felt exhibited less voltage loss at 80 mA cm^−2^ (Figure 8F). As a result, the assembled ZBFB achieves an energy efficiency of 82.5% at 80 mA cm^−2^.

Recently, boron doped graphene (BDG) has found to be promising for ZBFBs for its superior electrocatalytic activity, large surface area and electrical conductivity.^[^ ^59^ ^]^ BDG exhibited a less voltage loss (145 mV) compared to reduced graphene oxide (264 mV). The reduced voltage drop in BDG is an indication of the enhancement in the electrocatalytic activity over the reduced graphene oxide which improves the efficiency of the battery.

Surface defects with N doping and O functionalized groups were obtained through high temperature plasma treatment of graphite felt by Archana et al.^[^ ^60^ ^]^ This process enhanced the surface area of the graphite felt in addition with the N doping and O functionalization which promoted the kinetics of Br_2_/Br^−^ redox reaction. The overall Br_2_ in its complex limits the kinetics of the redox reaction. Therefore, this research has been proposed as a potential method to increase the electrochemical active surface area of the electrode materials along with the catalytic centers such as N and O. Maximum energy efficiency of 77% was obtained as compared to the graphite felt with doping and functionalization with low surface area.

#### 4.1.3 Composite and hybrid materials

Composite and hybrid materials have strong physical advantages over some other commercial non‐composite/non‐hybrid materials in terms of the superior mechanical flexibility and stability imparted by the composited structure. Polymers and metals are considered as suitable candidates to be composited or hybridized with the carbons because of their light weight, low cost, and flexibility. Composite and hybrid materials have been implemented for the development of ZBFBs in various ways. Suresh et al. introduced a nano porous carbon‐plastic composite electrode as the anode for ZBFBs.^[^ ^61^ ^]^ The electrode increased zinc utilization by 10% and resulted in a 17% increment in the coulombic efficiency. Further, the nano‐porous electrode was filled graphite which acted as a conductive network though the composite plate resulting in 50% zinc utilization and 20% increment of coulombic efficiency. Pt@Graphite Felt also has been used in the hope of improving the Br_2_/Br^−^ redox reaction.^[^ ^62^ ^]^ The noble Pt metal strongly improves the charge transfer between Pt@Graphite and bromine, which ensured maximum coulombic and energy efficiencies of ∼96%, ∼83%, at 300 cycles, respectively.

Williams et al. initiated metal oxide coating on carbon‐based electrodes for ZBFBs.^[^ ^63^ ^]^ The author reported hierarchical pores within a macro‐porous carbon nanofibre mat with large mesopores (>10 nm) and micropores ≤2 nm within the fibres. The hierarchical pores demonstrated improvements over activated carbon in coulombic efficiency as well as rate capability up to 30 mA/cm^2^. It was found that the metal oxide coatings enhanced rate capability and voltaic efficiency by adsorbing the bromine complex more efficiently.

Recently, Yin et al. introduced a tin (Sn)‐modified 3D carbon felt (Sn3D) as an anodic binder‐free host material for ZBFBs.^[^ ^64^ ^]^ In comparison to pristine carbon felt, the modified carbon ensured uniform Zn platting/stripping. The Sn‐modified carbon exhibited an average coulombic efficiency of 99.4% after 290 cycles.

Carbon and graphite felts composite have been commonly used as electrode materials in ZBFBs due to their large surface area, low cost, good stability and high porosity.^[^ ^65^ ^]^ The active carbon based slurry was dispersed in ZnBr_2_ solution and coated onto the carbon felt electrode.^[^ ^66^ ^]^ Further, the carbon felt was then put in the 6.0 m ZnBr_2_ solution for 30 s to achieve positive electrode (thickness of 3 mm). This cell obtained an energy efficiency of 82 % and columbic efficiency of 92% at 20 mA·cm^−2^.

### 4.2 Separators for ZBFBs

#### 4.2.1 Various separator materials for ZBFBs

Separators are used in ZBFBs to allow ionic exchange between the electrodes during cycling and also to avoid short circuits. There are various commonly used separators for ZBFBs. The SF600 membrane (produced by Asahi Kasei Co., Tokyo, Japan) is a porous separator with low bromine transport rate (3.4 × 10^−9^ mol Br_2_ cm^−2^ s^−1^) and resistivity (1.28 Ω cm).^[^ ^67^ ^]^ The microporous plastic polyolefins Daramic and Entek are other commonly used separators for ZBFBs.^[^ ^68^ ^]^

#### 4.2.2 Functional material modified separators for ZBFBs

The separator is a crucial component of ZBFBs which maintains the crossover of ions during charge/discharge. However, it also allows the passage of aqueous Br_2_ through its porous structure which cause self‐discharge. Over the years, little research has been carried out to stop the migration and diffusion of Br_2_ in ZBFBs. Coating the separator could be a promising way to control the diffusion of Br_2_ to the anode. In particular, a high surface area‐based carbon coating sandwiched between the two polypropylene separators could be an effective solution with the following advantages:

a) The high surface area carbon adsorbs the Br_2_ which reacts with the carbon and H_2_O to produce HBr, which can mitigate the pH issue in ZBFBs.^[^ ^69^ ^]^
b) The zinc dendrite which penetrates the separator will react with the adsorbed Br_2_ in the sandwiched carbon and produce ZnBr_2_. So, no short circuit will happen.
c) The self‐discharge will be reduced, as Br_2_ will mostly be adsorbed by the sandwiched carbon which won't allow the Br_2_ to react with the Zn.

To minimize the Br_2_ crossover, Kim et al. introduced Nafion‐filled 16 μm‐thick porous separator instead of 600 μm pristine SF‐600 separator.^[^ ^70^ ^]^ The sulfonate ion of Nafion filled the pores of the separator which maintained smaller resistance, and higher voltage efficiency leading to higher voltaic and energy efficiency. Nafion/PP showed two orders of magnitude less Br_2_ diffusivity (7.53 × 10^−9^ cm^−2^ min^−1^) than that of the SF‐600 (2.67 × 10^−7^ cm^−2^ min^−1^), clearly demonstrating the high Br_2_ blocking ability of the Nafion ionomer. Lee et al. demonstrated a 3D mesh interlayer to improve the electrochemical performance of ZBFBs.^[^ ^71^ ^]^ The modified ZBFB showed higher energy efficiency at 80 mA cm^−2^, which is surplus of 14.7 % over the contemporary polymer mesh. Recently, Naresh et al. introduced a MWCNT/PAN‐Daramic membrane with a significantly improved ability to inhibit bromine diffusion relative to the conventional microporous membrane.^[^ ^37^ ^]^ PAN and Daramic membrane, respectively introduced only 68.53 % and 71.2 % efficiencies at 160 mA cm^−2^. On the other hand, the MWCNT/PAN composite Daramic membrane displayed an extraordinary long cycling performance with high coulombic efficiencies of >97%.

### 4.3 Electrolyte additives and their functions in ZBFBs

Smooth and uniform Zn plating during the charging process is a crucial requirement for ZBFBs. The morphology of the zinc plating significantly affects the battery performance (e.g., coulombic efficiency, voltaic efficiency, longevity of the battery).^[^ ^72^ ^]^ The Zn plating is adversely affected by some electrochemical reactions including dendrite formation, HER and corrosion reactions in ZBFBs. Electrolyte additives are commonly used in ZBFBs to improve the electrolyte conductivity and to reduce the possible undesired side reactions to improve the electrochemical performance of the battery. Some additives act to fix the liquid bromine in the bromine half‐cell to avoid self‐discharge in ZBFBs and assist to mitigate the H_2_ evolution. As a routine practice, electrolyte additives are mostly used to improve the zinc deposition by inhibiting the formation of dendrites and preventing the HER.^[^ ^73^ ^]^ Different additives like organic molecules, polymers, and metal ions have been incorporated into electrolytes for the inhibition of Zn dendritic problems in ZBFBs. Both polymers and organic molecules can be adsorbed onto the protruding parts formed on the electrode surface, blocking the access to various locations of the anode surface.^[^ ^74^ ^]^ Consequently, it is more likely that organic molecules will cover protruding parts and slow down zinc deposition. Although the adsorption of polymeric and organic additives improves with increasing polarity of these additives, exceeding the optimum concentration level can lead to adverse effects in the electrochemical process of Zn plating.^[^ ^74^ ^]^ The need for suppressing dendrite growth can lead to significant improvement of Zn‐bromine flow‐battery performance.

#### 4.3.1 Polymers as additives

Adding polymers to electrolytes plays a crucial role in the morphology of Zn anodes by suppressing Zn dendrites and side reactions in zinc‐bromine flow batteries. Polymers not only function to reduce of to reduce the dendrite nucleation sites on Zn electrode surfaces but also decrease the water content of soluble Zn‐based compounds to avoid any HER reactions. Binders are adsorbed onto the electrode surface, can regulate the distribution of local current near the protuberances of a Zn anode. Therefore, the polarisation of the electrode surface reduces, leading to uniform current distribution and even morphology through a lower reduction rate of Zn deposition. A wide range of polymers have been used to control zinc plating, such as polyvinyl alcohol, polyethylene glycol, and polyethyleneimine.^[^ ^75^ ^]^ Despite the study of a variety of promising polymeric‐additive candidates, there is still much room left for different systematic investigations and discovery of optimal concentrations of the polymeric additives along with long‐term and real cycling tests.

#### 4.3.2 Organic molecules

Zn electrode surfaces can be also improved by various additives of organic molecules such as molecular surfactants and room‐temperature ionic liquids (e.g., alkyl imidazoles, alkyl pyridines, quaternary ammonium salts, quaternary phosphorus salts, pyrroles, and terbium salts). For example, polyoxyethylene (20) sorbitan monolaurate (Tween 20) is an additive that has been introduced to improve the ZBFBs performance. Small addition of Tween 20 significantly improved the coulombic efficiency in comparison to the conventional electrolyte.^[^ ^76^ ^]^ These additives are able to control the deposition morphology leading to a uniform surface without dendrites.^[^ ^72a^ ^]^ In general, the more polar the organic additive, the stronger its adsorption onto a Zn anode.^[^ ^74^ ^]^ However, zinc deposition will be severely polarised by excessively strong adsorption.^[^ ^74^ ^]^ Recently, tetrapropylammonium bromide and tetrabutylammonium bromide have displayed excellent bromine fixation ability to reduce the diffusion of liquid Br_2_ in ZBFBs.^[^ ^77^ ^]^ Xu et al. introduced a novel bromine‐fixed additive, tetraethylammonium bromide (TEAB), which is low‐cost and proposed to slow the diffusion of Br_2_ in ZBFBs and enhanced the capacity retention by 30% as compared to the MEP complexing agent.^[^ ^78^ ^]^ Zeon et al. employed zinc perchlorate as an effective additive, resulting highly stable ZBFBs.^[^ ^67^ ^]^ In the presence of zinc perchlorate additives, the ZBFB exhibited superior coulombic and energy efficiency in comparison to pristine and zinc chloride.

#### 4.3.3 Ionic liquid additives/complexing agents

Ionic liquids are commonly used in the electrolyte of ZBFBs to obtain quality zinc electrodeposition during the charging process. Rajarathnam et al. demonstrated six ionic liquids in contrast to the conventional BSA MEP to understand their influence on the Zn plating during charging in ZBFBs.^[^ ^23a^ ^]^ Among the various complexing agents, the best performing ionic liquid was found to be [C_2_Py] Br, which had lower polarisation resistance and exhibited higher exchange currents than the electrolytes with other additives.

#### 4.3.4 Other functional additives

**FIGURE 9:**
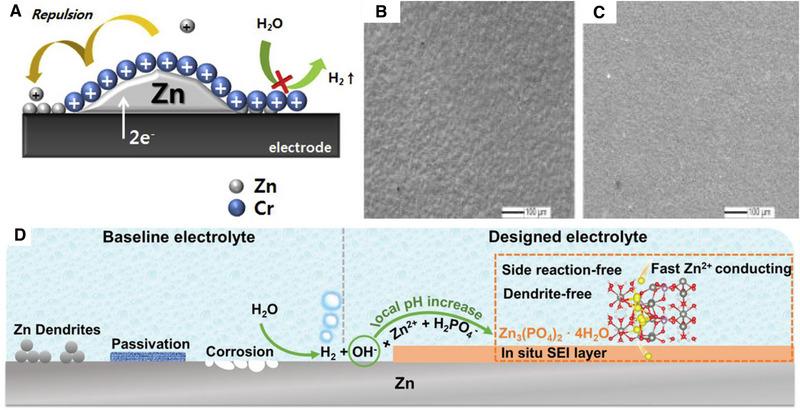
(A) Mechanism to control the pH using Cr^3+^ secret agent by the electrostatic repulsion (taken and modified from other works). Reproduced with permission.^[^ ^34^ ^]^ Copyright 2018, Elsevier Ltd. (B) 10^−4^ m Pb and (C) 10^−4^ m Pb + 5 × 10^−5^ m tetrabutylammonium bromide (TBAB). Reproduced with permission.^[^ ^79^ ^]^ Copyright 2011, Elsevier Ltd. (D) Illustration of the possible reactions and SEI formation mechanism on Zinc electrode surface. Reproduced with permission.^[^ ^81^ ^]^ Copyright 2021 John Wiley & Sons, Inc.

Cr^3+^ functionalized additive was proposed to minimize the zinc dendrite and HER issue in ZBFB.^[^ ^34^ ^]^ It was suggested that the Cr^3+^ is able to form a positively charged electrostatic shield surrounding the zinc seeds without deposition. This electrostatic shield repels H^+^ approaching the zinc seeds and protect zinc from HER (Figure 9A). Tetrabutylammonium bromide (TBAB) as another potential additive has been testified to achieve smooth zinc plating in ZBFBs. It was discovered that the dual addition of 10^−4^ m Pb (II) and 5 × 10^−5^ m TBAB introduces more effective suppression on mossy zinc growth (Figure 9B,C).^[^ ^9^, ^79^ ^]^

Zinc metal is thermodynamically unstable in acidic and alkaline conditions and dissolves releasing H_2_ gas.^[^ ^80^ ^]^ The H_2_ generation increases the electrolyte pH and continuous Zn consumption by producing detrimental compound Zn(OH)_2_/ZnO.^[^ ^16b^ ^]^ Zeng et al. suggested that 140 nm thick SEI layer enables uniform dendrite‐free Zn deposition and reduces the side reactions via isolating active Zn metal from the bulk electrolyte.^[^ ^81^ ^]^ The authors demonstrated Zn(H_2_PO_4_)_2_ as a potential corrosion mediator by creating a thin SEI layer on the zinc (Figure 9D).

Grimes et al. invented a method combining H_2_ and Br_2_ in the presence of a catalyst.^[^ ^29^ ^]^ To prevent the solid Zn (OH)_2_ formation and other issues of ZBFBs, it was suggested that ruthenium as a catalyst is potentially capable of forming acid (HBr) before it can lead to the aforementioned solid species. The gaseous decomposition of hydrogen reacts with bromine in the presence of a catalyst which encourages the formation of hydrogen and bromide ions.

### 4.4 Static Zn‐Br battery

**FIGURE 10:**
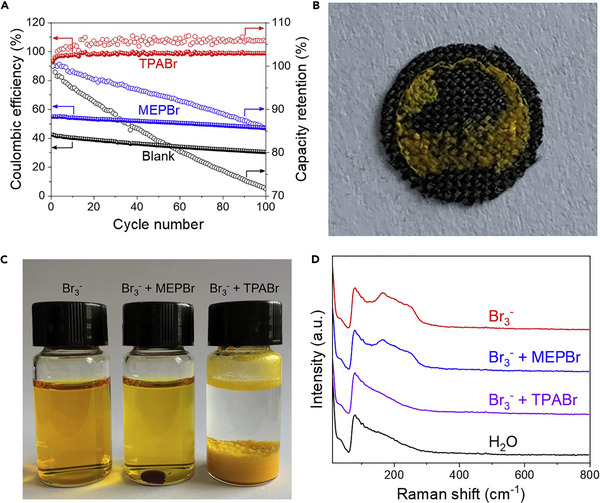
(A) Cycling performance of the static cells with different electrolytes at 0.3 mA cm^−2^. (B) Optical image of the carbon electrode surface after being charged for 1 h at 5 mA cm^−2^ in TPABr electrolyte. (C) Br_3_^−^ solution and its mixture with different complexing agents. (D) Raman spectra of the Br_3_^−^ solution and its mixtures with MEPBr and TPABr. Reproduced with permission.^[^ ^77^ ^]^ Copyright 2020, Elsevier Ltd.

Apart from the typical ZBFBs, the non‐flow battery is also an area of great research interest. In the ZBFB, the pumps are driven by the battery, so some energy efficiency is sacrificed because of the energy required. The flow of electrolyte over the surface of the electrode is also somewhat responsible for degradation of the electrode over time in the flow battery. In the non‐flow battery, no pumps are required which saves energy and cost by reducing the requirement for auxiliary parts associated with the pumps. The non‐flow battery also overcomes the concentration over‐potential which is the results of the concentration gradient between bulk electrolyte and electrode surface.^[^ ^82^ ^]^. Recently, researchers have also focused on the non‐flow ZBM with and without separator. Gao et al. demonstrated a zinc bromine static battery with a glass fibre membrane as the separator to control the self‐discharge and improve the energy efficiency (Figure 10).^[^ ^77^ ^]^ This static battery was achieved by using tetrapropylammonium bromide (TPABr) as the complexing agent. The complexation from TPABr is physically confined in the porous carbon to decrease the crossover diffusion of Br_2_ and self‐discharge. The following figures in Figure 10 show the advantages of the TPABr complexing agent over the typically used MEPBr in ZBFBs where the TPABr is outperforming the MEPBr in terms of coulombic efficiency and capacity retention.

**FIGURE 11:**
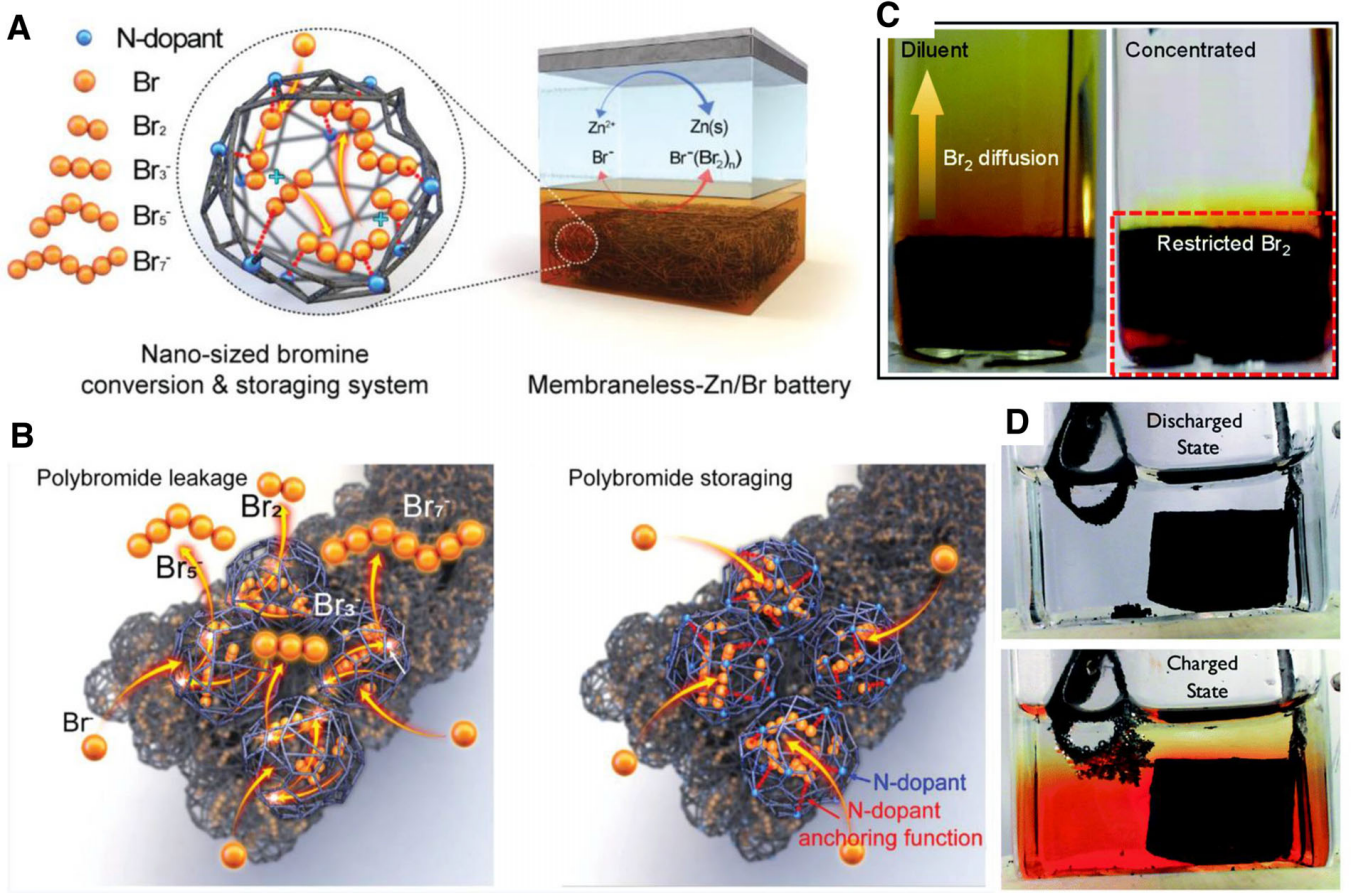
(A) Schematics of the proposed NGF electrode and mechanism for Br conversion/storage in membraneless‐Zn/Br battery. (B) Mechanism illustration of NGF‐1000 and NGF‐700 electrodes for the capture and storage of Br species. Reproduced with permission.^[^ ^57^ ^]^ Copyright 2019 John Wiley & Sons, Inc. (C) 2.5 m ZnBr_2_ and 20 m ZnBr_2_ + 10 m LiCl electrolyte after charging process. Reproduced with permission.^[^ ^83^ ^]^ Copyright 2022, The Royal Society of Chemistry. (D) charge and discharge state of membrane free static Zinc bromine battery. Reproduced with permission.^[^ ^84^ ^]^ Copyright 2017, The Royal Society of Chemistry.

Lee et al. demonstrated a non‐flow zinc bromine battery without a membrane.^[^ ^57^ ^]^ The nitrogen (N)‐doped microporous graphene felt (NGF) was used as the positive electrode (Figure 11A,B). The NGF electrode could efficiently capture Br^−^ and polybromide anions with the abundant nitrogen dopant sites and facilitate the redox conversion reactions through an electrochemical chemical growth mechanism. The NGF electrode with separator enabled a long‐term cycling (1000 cycles), with an energy efficiency over 80%. Also, the confined conversion reactions of the concentrated bromide/polybromide within N‐doped graphene on graphite felt (NGF) leads to fast charge/discharge and low self‐discharge as demonstrated in Figure 11A.

Recently, Liu et al. introduced an alternative type of membrane‐free zinc bromine static battery (Figure 11C).^[^ ^83^ ^]^ The authors proposed the use of different concentration of electrolytes for this research and a highly concentrated electrolyte showed better performance in the presence of a membrane. The liquid Br_2_ diffusion was mitigated in the highly concentrated electrolyte whereas the diluted electrolyte exhibited higher coulombic efficiency loss. Biswas et al. also reported a membrane‐free zinc bromine static battery (Figure 11D).^[^ ^84^ ^]^ The anode was placed near the aqueous region of the electrolyte to avoid self‐discharge. This membrane‐free design saw cycling stability for over 1000 cycles with high coulombic efficiency (90%) and energy efficiency (60%). A device with an energy density of nearly 9 Wh L^−1^ and a cost of $100 per kWh at scale was achieved.

## 5 CONCLUSION AND FUTURE RECOMMENDATION

Over the years extensive research has been carried out in ZBFBs. Various electrode materials and electrolyte modifications have been explored to improve the electrochemical performance of ZBFBs. In this review, the major technical issues of ZBFBs and recent progress on the development of functional materials and additives to overcome these challenges have been discussed. Based on the review, the authors of this manuscript recommend the following.

(1) From the literature it is clear that zinc corrosion and HER are the fundamental issues of ZBFBs along with the formation of zinc dendrites. The corrosion and HER cause pH rise and deteriorate the ZBFBs performance in terms of efficiency and longevity. However, very limited studies have been conducted on this technical issue of ZBFBs. In this review, we have highlighted these issues based on available literature and propose some mitigation steps so that more research will be devoted in this topic to make ZBFBs more realistic in future.
(2) Considering the recent research advances on other Zinc‐based rechargeable batteries, many strategies could be adopted to mitigate those issues in ZBFBs. For example, new additives could be developed to control the corrosion and HER reaction to maintain smooth zinc plating during charging. To control the pH rise in ZBFBs, a strong buffer could be an effective solution to optimize the longevity and overall performance of ZBFBs. Carbon materials doped with different heteroatoms can be useful to promote the zinc plating/depleting due to their higher electronic conductivity. New complexing agents and different conductive catalysts could be developed and introduced in the positive electrode to accelerate the Br_2_ complexation and decomplexation process during cycling.
(3) Some critical parameters are missing in most of the literature reports such as the density of active carbon on the positive and negative electrode (mg·cm^−2^), the electrolyte recipe, the volume of electrolyte used. All of these are critical parameters to evaluate the electrode electrochemical performance, volumetric and gravimetric energy density of ZBFBs.
(4) The negative and positive electrodes can be designed as follows to achieve good electrochemical performance form ZBFBs: 1) the negative electrode materials need to be highly conductive with low surface area with optimized adhesion of the zinc to plate and dissolve optimally. High surface area materials are less desirable as the zinc can penetrate into the surface pores and won't be smoothly depleted during the discharge. 2) The positive electrode materials need to be highly conductive and high surface area carbon with suitable porosity. Heteroatom doping or catalysts could be effective in the positive electrode to promote the Br_2_ complex reaction.
(5) New battery configurations based on Zn‐Br chemistry such as static Zn‐Br cells with liquid or solid electrolytes have been reported and are worthy of further exploration, although some key parameters like energy density and cycling stability still need to be improved. The separator coating on the cathode side could be useful to moderate the pH issue and dendrite growth in both static and flow ZBBs. Integrated systems such as solar rechargeable ZBFBs might be a promising direction to make better use of the renewable energy in the future.
(6) Currently, separators produced by Daramic, Ashi, and Entek are commonly used for zinc‐bromine flow battery systems. These separators are bulky, and the pores are significantly larger than the Br_2_ molecules. New polymer‐based separators with small pores and less weight would be useful for commercial application.

## CONFLICT OF INTEREST STATEMENT

The authors declare no conflict of interest.
